# Supplementary material for: Genome-wide association studies of COVID-19 vaccine seroconversion and breakthrough outcomes in UK Biobank
Source: Nat Commun. 2024 Oct 9;15:8739. doi: 10.1038/s41467-024-52890-6 (PMC11464770; doi:10.1038/s41467-024-52890-6)
Supplement: Supplementary file 1 — Supplementary information [file 41467_2024_52890_MOESM1_ESM.pdf]

Supplementary information

Supplementary Note 1. ICD-10 codes definition.

The International Classification of Diseases (ICD) 10th Revision code is used to identify the coded clinical entry. In particular:

- U07.1: COVID-19, virus identified.
  Code used when COVID-19 has been confirmed by laboratory testing.
- U07.2: COVID-19, virus not identified.
  Code used when COVID-19 is diagnosed clinically or epidemiologically, but without laboratory testing.

Supplementary Figure 1. Histogram showing the number of days between the last vaccination and the antibody test. The red line indicates the mean value. **(A)** Days between the first vaccine dose and the antibody test, with a mean of 38.4 days. **(B)** Days between the second vaccine dose and the antibody test, with a mean of 24.7 days.

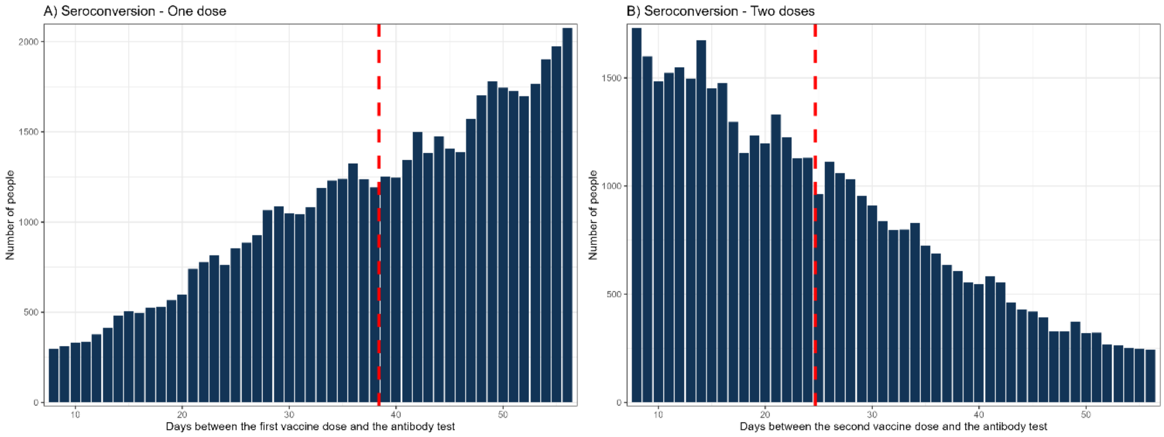

Supplementary Figure 2. Flow chart of the quality control performed by PLINK2 on genotype calls and imputed variants. **(A)** Flow chart for seroconversion - one dose cohort. **(B)** Flow chart for seroconversion - two dose cohort. **(C)** Flow chart for breakthrough infection. **(D)** Flow chart for breakthrough susceptibility.

A)

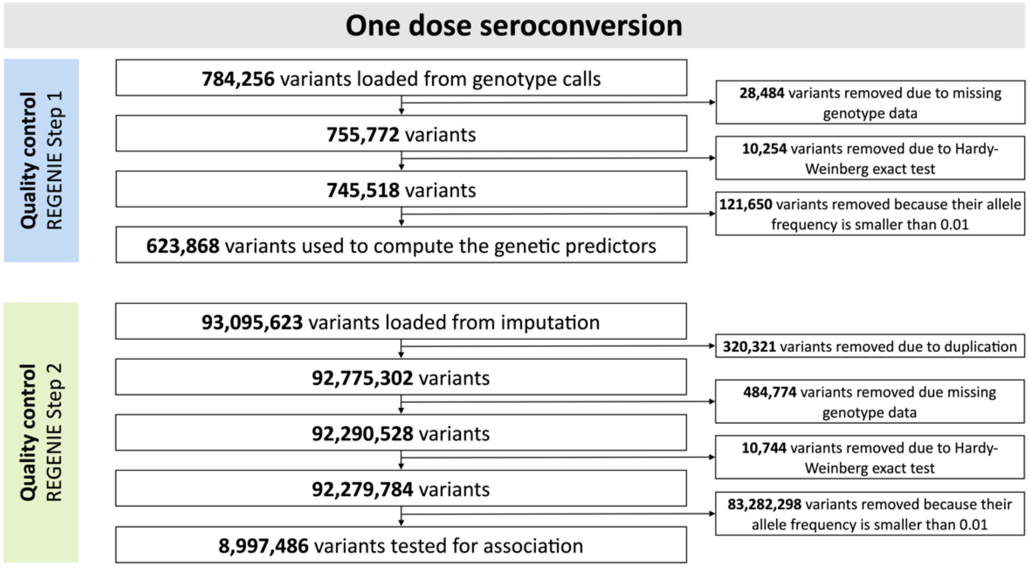

B)

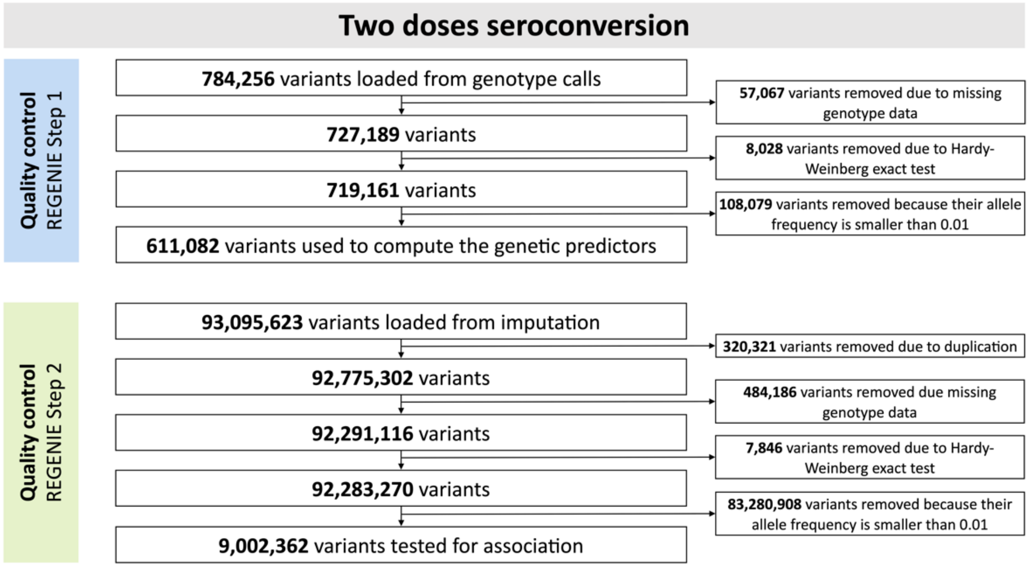

C)

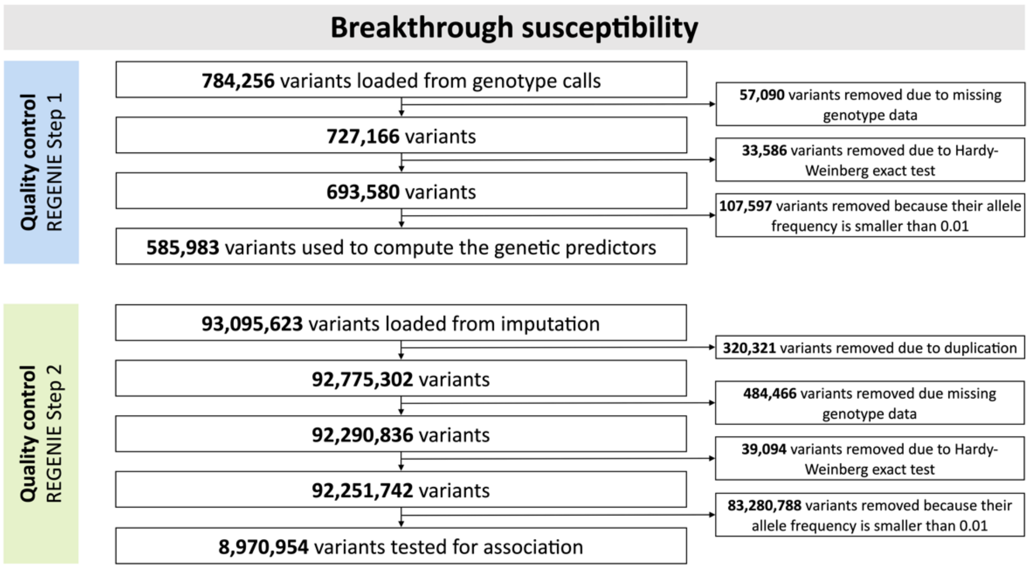
Breakthrough susceptibility
Quality control
REGENIE Step 1
784,256 variants loaded from genotype calls
57,090 variants removed due to missing genotype data
727,166 variants
33,586 variants removed due to Hardy-Weinberg exact test
693,580 variants
107,597 variants removed because their allele frequency is smaller than 0.01
585,983 variants used to compute the genetic predictors
Quality control
REGENIE Step 2
93,095,623 variants loaded from imputation
320,321 variants removed due to duplication
92,775,302 variants
484,466 variants removed due missing genotype data
92,290,836 variants
39,094 variants removed due to Hardy-Weinberg exact test
92,251,742 variants
83,280,788 variants removed because their allele frequency is smaller than 0.01
8,970,954 variants tested for association

D)

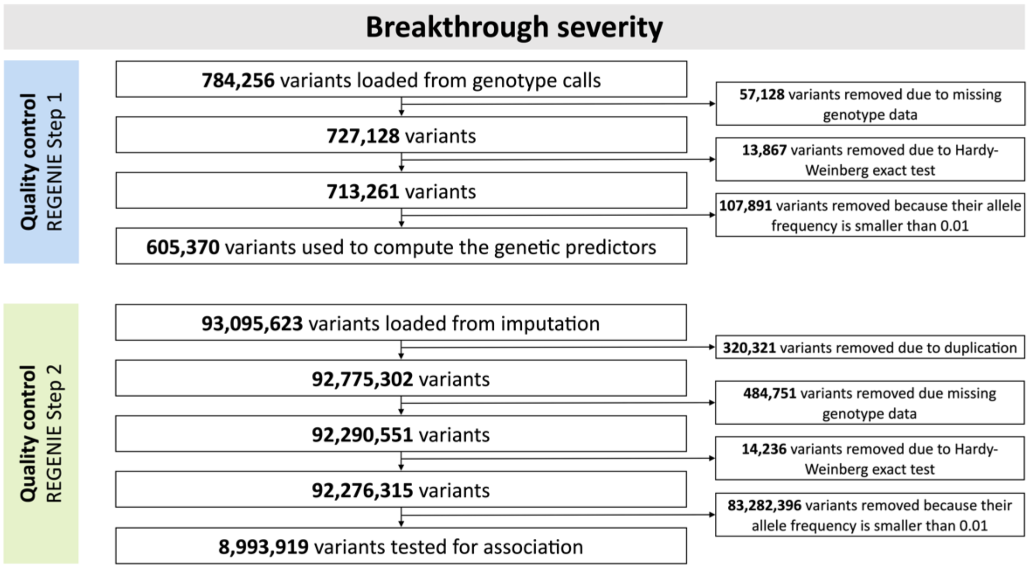
Breakthrough severity
Quality control
REGENIE Step 1
784,256 variants loaded from genotype calls
57,128 variants removed due to missing genotype data
727,128 variants
13,867 variants removed due to Hardy-Weinberg exact test
713,261 variants
107,891 variants removed because their allele frequency is smaller than 0.01
605,370 variants used to compute the genetic predictors
Quality control
REGENIE Step 2
93,095,623 variants loaded from imputation
320,321 variants removed due to duplication
92,775,302 variants
484,751 variants removed due missing genotype data
92,290,551 variants
14,236 variants removed due to Hardy-Weinberg exact test
92,276,315 variants
83,282,396 variants removed because their allele frequency is smaller than 0.01
8,993,919 variants tested for association

Supplementary Table 1. Population characteristics of each one of the cohorts. For seroconversion analyses, age was calculated at time of antibody testing. For the breakthrough analyses, age was measured at time of receiving the first COVID-19 vaccine dose. Index of multiple deprivation measures the relative levels of poverty. The lower the value, the more deprived the area is.

Variables		Seroconversion		Breakthrough	
		One dose	Two doses	Susceptibility	Severity
N		53,207	42,509	315,323	74,662
**Baseline characteristics**					
Sex (%)	Female	29,284 (55.0)	24,590 (57.8)	173,864 (55.1)	40,959 (54.9)
	Male	23,923 (45.0)	17,919 (42.2)	141,459 (44.9)	33,703 (45.1)
Age (Mean (SD))		66.04 (7.77)	71.53 (6.18)	69.64 (7.94)	67.72 (8.05)
Body mass index (Mean (SD))		26.53 (4.39)	27.11 (4.51)	27.30 (4.68)	27.27 (4.69)
**Indices of multiple deprivation**					
Index of multiple deprivation (Mean (SD))		14.05 (11.50)	14.96 (12.17)	16.45 (13.20)	15.83 (12.61)
**Ethnic background**					
British		53,207 (100)	42,509 (100)	315,323 (100)	74,662 (100)

Supplementary Table 2. GWAS results for the four different traits. Top lead independent variants (P-Value $\leq 5 \cdot 10^{-8}$, $r^2 \leq 0.1$ and window = 250kb) and lead independent variants of each locus (P-Value $\leq 5 \cdot 10^{-8}$ and $r^2 \leq 0.1$) identified in FUMA are noted. Association was tested by logistic regression in REGENIE. **Note:** SNP = Single nucleotide polymorphism; CHR = Chromosome; BP = Base pair; EAF = Effect allele frequency; EA = Effect allele; OA = Other allele; OR = Odds Ratio; SE = Standard error; PVAL = P-Value.

Type of variant	Genomic locus	Phenotype	SNP	CHR	BP	EA	OA	EAF	OR	SE	PVAL	Function	Gene/Nearest genes
Top lead variant	1	Seroconversion - One dose	rs9275109	6	32,649,676	T	G	0.61	0.86	0.01	1.1e-26	intergenic	HLA-DQB1 - MTCO3P1
Lead variant	1	Seroconversion - One dose	rs3134931	6	32,190,620	C	T	0.30	0.92	0.02	1.4e-08	intronic	NOTCH4
Lead variant	1	Seroconversion - One dose	rs7754570	6	32,397,190	G	C	0.03	1.26	0.04	2.6e-08	intergenic	BTNL2 - HLA-DRA
Lead variant	1	Seroconversion - One dose	rs2395195	6	32,447,989	A	G	0.13	0.86	0.02	5.1e-12	intergenic	HLA-DRB9 - HLA-DRB5
Lead variant	1	Seroconversion - One dose	rs146281128	6	32,528,207	C	T	0.01	0.67	0.07	3.8e-08	upstream	HLA-DRB6
Lead variant	1	Seroconversion - One dose	rs145945003	6	32,574,956	G	A	0.03	1.32	0.04	6.5e-14	intergenic	HLA-DRB1 - HLA-DQA1
Lead variant	1	Seroconversion - One dose	rs28802989	6	32,607,019	G	C	0.15	0.84	0.02	3.7e-19	UTR3	HLA-DQA1
Lead variant	1	Seroconversion - One dose	rs4713558	6	32,607,055	C	T	0.05	0.81	0.03	1.3e-09	UTR3	HLA-DQA1
Lead variant	1	Seroconversion - One dose	rs1794514	6	32,667,473	C	G	0.25	1.11	0.02	6.4e-11	intergenic	HLA-DQB1 - MTCO3P1
Lead variant	1	Seroconversion - One dose	rs3129733	6	32,685,456	G	A	0.17	0.90	0.02	2.1e-08	upstream	XXbac-BPG254F23.7
Lead variant	1	Seroconversion - One dose	rs2071472	6	32,784,620	T	C	0.27	0.90	0.02	4.8e-11	intronic	HLA-DOB - TAP2
Lead variant	1	Seroconversion - One dose	rs3130216	6	33,077,271	A	G	0.54	0.92	0.01	6.0e-09	intergenic	COL11A2P1 - HLA-DPB2
Top lead variant	2	Seroconversion - One dose	rs79510369	2	160,858,048	T	A	0.01	1.48	0.06	5.3e-10	intronic	PLA2R1
Top lead variant	1	Seroconversion - Two dose	rs68033958	6	32,634,226	A	G	0.16	0.82	0.02	1.4e-21	intronic	HLA-DQB1
Lead variant	1	Seroconversion - Two dose	rs28752480	6	31,574,865	C	A	0.07	1.23	0.03	1.1e-11	intergenic	NCR3 - UQCRHP1
Lead variant	1	Seroconversion - Two dose	rs2261033	6	31,603,591	G	A	0.43	0.91	0.02	1.1e-08	intronic	PRRC2A
Lead variant	1	Seroconversion - Two dose	rs732162	6	32,394,913	A	G	0.33	1.10	0.02	1.9e-08	intergenic	BTNL2 - HLA-DRA
Lead variant	1	Seroconversion - Two dose	rs28383322	6	32,592,796	T	C	0.20	1.15	0.02	1.8e-11	intergenic	HLA-DRB1 - HLA-DQA1
Lead variant	1	Seroconversion - Two dose	rs9275766	6	32,689,362	G	A	0.09	1.18	0.03	3.8e-09	intergenic	XXbac-BPG254F23.7 - HLA-DQB3
Top lead variant	2	Seroconversion - Two dose	rs3094055	6	30,332,146	G	C	0.77	0.89	0.02	2.1e-10	upstream	UBQLN1P1
Top lead variant	1	Breakthrough susceptibility	rs73062389	3	45,835,417	A	G	0.06	1.22	0.01	4.0e-56	intronic	SLC6A20
Lead variant	1	Breakthrough susceptibility	rs141045534	3	45,637,109	C	T	0.01	1.18	0.03	3.5e-08	exonic	LIMD1
Lead variant	1	Breakthrough susceptibility	rs59776512	3	45,835,415	GA	G	0.06	1.10	0.01	7.1e-16	intronic	SLC6A20
Lead variant	1	Breakthrough susceptibility	rs2531757	3	45,850,520	A	C	0.34	1.04	0.01	3.7e-09	intergenic	SLC6A20 - LZTFL1
Lead variant	1	Breakthrough susceptibility	rs6789386	3	46,174,395	C	G	0.67	1.04	0.01	2.7e-08	intergenic	XCR1 - FLT1P1
Top lead variant	2	Breakthrough susceptibility	rs16861415	3	186,696,364	C	T	0.08	0.84	0.01	6.8e-55	intronic	ST6GAL1
Lead variant	2	Breakthrough susceptibility	rs71322420	3	186,621,318	G	C	0.19	0.94	0.01	1.2e-15	intergenic	RPS20P14 - RP11-42D20.1
Lead variant	2	Breakthrough susceptibility	rs13097481	3	186,700,792	C	A	0.47	1.05	0.01	6.0e-13	intronic	ST6GAL1
Lead variant	2	Breakthrough susceptibility	rs114664200	3	186,725,011	C	G	0.02	0.87	0.02	5.6e-09	intronic	ST6GAL1
Top lead variant	3	Breakthrough susceptibility	rs11673136	19	9,007,748	G	A	0.48	1.08	0.01	4.6e-34	intronic	MUC16
Top lead variant	4	Breakthrough susceptibility	rs112313064	19	5,831,724	C	T	0.36	1.06	0.01	1.1e-22	exonic	FUT6
Lead variant	4	Breakthrough susceptibility	rs3760775	19	5,841,356	T	G	0.06	1.08	0.01	3.6e-09	intergenic	FUT6 - FUT3
Top lead variant	5	Breakthrough susceptibility	rs681343	19	49,206,462	T	C	0.50	0.95	0.01	1.4e-17	exonic	FUT2
Top lead variant	6	Breakthrough susceptibility	rs1977829	10	111,975,041	A	G	0.18	0.94	0.01	1.4e-12	intronic	MXI1
Top lead variant	7	Breakthrough susceptibility	rs2550250	3	195,500,549	T	C	0.44	1.05	0.01	1.8e-12	intronic	MUC4
Top lead variant	8	Breakthrough susceptibility	rs6676150	1	155,123,837	C	G	0.39	1.04	0.01	2.7e-12	intergenic	HMGN2P18 - KRTCAP2
Top lead variant	9	Breakthrough susceptibility	rs17347644	3	101,547,733	T	C	0.35	0.96	0.01	1.3e-10	intronic	NFKBIZ
Top lead variant	10	Breakthrough susceptibility	rs5117	19	45,418,790	C	T	0.24	0.96	0.01	4.7e-09	intronic	APOC1
Top lead variant	1	Breakthrough severity	rs429358	19	45,411,941	C	T	0.15	1.21	0.03	1.1e-08	exonic	APOE

Supplementary Table 3. Population characteristics of the validation cohort. For seroconversion analyses, age was calculated at time of antibody testing. For the breakthrough analyses, age was measured at time of receiving the first COVID-19 vaccine dose. Index of multiple deprivation measures the relative levels of poverty. The lower the value, the more deprived the area is.

Variables		Seroconversion		Breakthrough	
		One dose	Two doses	Susceptibility	Severity
N		8,189	6,533	57,851	12,727
**Baseline characteristics**					
Sex (%)	Female	4,679 (57.1)	3,932 (60.2)	32,494 (56.2)	7,160 (56.3)
	Male	3,510 (42.9)	2,601 (39.8)	25,357 (43.8)	5,567 (43.7)
Age (Mean (SD))		64.24 (7.57)	70.07 (6.75)	67.32 (8.22)	65.76 (8.03)
Body mass index (Mean (SD))		26.35 (4.49)	27.02 (4.68)	27.31 (4.83)	27.23 (4.85)
**Indices of multiple deprivation**					
Index of multiple deprivation (Mean (SD))		16.00 (12.75)	17.34 (13.26)	20.28 (14.95)	18.79 (14.12)
**Ethnic background**					
White (%)	White	46 (0.6)	42 (0.6)	337 (0.6)	59 (0.5)
	British	2,817 (34.4)	2,219 (34.0)	16,147 (27.9)	3,974 (31.2)
	Irish	1,433 (17.5)	1,195 (18.3)	9,143 (15.8)	2,087 (16.4)
	Any other white background	2,090 (25.5)	1,538 (23.5)	11,515 (19.9)	2,698 (21.2)
Mixed (%)	Mixed	2 (0.0)	3 (0.0)	24 (0.0)	4 (0.0)
	White and Black Caribbean	66 (0.8)	48 (0.7)	450 (0.8)	113 (0.9)
	White and Black African	37 (0.5)	26 (0.4)	283 (0.5)	80 (0.6)
	White and Asian	108 (1.3)	72 (1.1)	630 (1.1)	154 (1.2)
	Any other mixed background	132 (1.6)	69 (1.1)	748 (1.3)	171 (1.3)
Asian or Asian British (%)	Indian	347 (4.2)	378 (5.8)	4,680 (8.1)	887 (7.0)
	Pakistani	68 (0.8)	47 (0.7)	1,240 (2.1)	201 (1.6)
	Bangladeshi	7 (0.1)	5 (0.1)	163 (0.3)	23 (0.2)
	Any other Asian background	92 (1.1)	87 (1.3)	1,386 (2.4)	255 (2.0)
Black or Black British (%)	Caribbean	189 (2.3)	181 (2.8)	3,015 (5.2)	578 (4.5)
	African	105 (1.3)	107 (1.6)	2,280 (3.9)	332 (2.6)
	Any other Black background	5 (0.1)	6 (0.1)	84 (0.1)	17 (0.1)
Chinese (%)		188 (2.3)	90 (1.4)	1,144 (2.0)	213 (1.7)
Other ethnic group (%)		292 (3.6)	254 (3.9)	3,202 (5.5)	618 (4.9)
Do not know (%)		17 (0.2)	15 (0.2)	143 (0.2)	21 (0.2)
Prefer not to answer (%)		148 (1.8)	147 (2.3)	1188 (2.1)	232 (1.8)

Supplementary Table 4. Validation results for the variants associated with each one of the traits. Validation cohort is non-European ancestry in UK Biobank. SNPs in red were validated. SNPs in bold refer to the top lead variants identified in FUMA. P-Value (validation) threshold to determine statistical significance was set to 0.05. Association was tested by logistic regression in REGENIE.

Type of variant	Genomic locus	Phenotype	SNP	EA	Main analysis				Validation			
					N	EAF	OR	P Value	N	EAF	OR	P Value
Top lead variant	1	Seroconversion - One dose	rs9275109	T	53,097	0.61	0.86	1.1e-26	8,170	0.65	0.84	1.5e-05
Lead variant	1	Seroconversion - One dose	rs3134931	C	53,109	0.30	0.92	1.4e-08	8,177	0.34	0.95	2.0e-01
Lead variant	1	Seroconversion - One dose	rs7754570	G	53,087	0.03	1.26	2.6e-08	8,172	0.03	1.27	1.8e-02
Lead variant	1	Seroconversion - One dose	rs2395195	A	52,808	0.13	0.86	5.1e-12	8,117	0.19	0.85	1.0e-03
Lead variant	1	Seroconversion - One dose	rs146281128	C	52,948	0.01	0.67	3.8e-08	8,120	0.03	0.73	5.9e-03
Lead variant	1	Seroconversion - One dose	rs145945003	G	52,670	0.03	1.32	6.5e-14	8,066	0.04	1.06	5.3e-01
Lead variant	1	Seroconversion - One dose	rs28802989	G	52,682	0.15	0.84	3.7e-19	8,044	0.19	0.83	2.3e-04
Lead variant	1	Seroconversion - One dose	rs4713558	C	52,794	0.05	0.81	1.3e-09	8,053	0.07	0.87	6.8e-02
Lead variant	1	Seroconversion - One dose	rs1794514	C	51,623	0.25	1.11	6.4e-11	7,918	0.20	1.02	6.6e-01
Lead variant	1	Seroconversion - One dose	rs3129733	G	52,878	0.17	0.90	2.1e-08	8,140	0.17	0.97	5.4e-01
Lead variant	1	Seroconversion - One dose	rs2071472	T	53,109	0.27	0.90	4.8e-11	8,177	0.30	0.90	7.4e-03
Lead variant	1	Seroconversion - One dose	rs3130216	A	53,109	0.54	0.92	6.0e-09	8,177	0.58	0.98	6.1e-01
Top lead variant	2	Seroconversion - One dose	rs79510369	T	53,068	0.01	1.48	5.3e-10	8,157	0.03	1.10	4.5e-01
Top lead variant	1	Seroconversion - Two dose	rs68033958	A	42,037	0.16	0.82	1.4e-21	6,497	0.20	0.86	3.3e-03
Lead variant	1	Seroconversion - Two dose	rs28752480	C	42,394	0.07	1.23	1.1e-11	6,520	0.06	1.02	8.3e-01
Lead variant	1	Seroconversion - Two dose	rs2261033	G	42,439	0.43	0.91	1.1e-08	6,532	0.45	0.96	2.8e-01
Lead variant	1	Seroconversion - Two dose	rs732162	A	42,418	0.33	1.10	1.9e-08	6,523	0.34	1.03	4.4e-01
Lead variant	1	Seroconversion - Two dose	rs28383322	T	39,725	0.20	1.15	1.8e-11	6,078	0.20	1.09	1.1e-01
Lead variant	1	Seroconversion - Two dose	rs9275766	G	42,439	0.09	1.18	3.8e-09	6,532	0.08	1.00	9.5e-01
Top lead variant	2	Seroconversion - Two dose	rs3094055	G	42,439	0.77	0.89	2.1e-10	6,532	0.80	0.98	6.7e-01
Top lead variant	1	Breakthrough susceptibility	rs73062389	A	314,757	0.06	1.22	4.0e-56	58,037	0.05	1.26	4.2e-12
Lead variant	1	Breakthrough susceptibility	rs141045534	C	313,756	0.01	1.18	3.5e-08	57,814	0.01	1.20	2.2e-02
Lead variant	1	Breakthrough susceptibility	rs59776512	GA	313,859	0.06	1.10	7.1e-16	57,761	0.07	1.07	1.7e-02
Lead variant	1	Breakthrough susceptibility	rs2531757	A	302,422	0.34	1.04	3.7e-09	53,729	0.30	1.04	1.9e-02
Lead variant	1	Breakthrough susceptibility	rs6789386	C	307,460	0.67	1.04	2.7e-08	55,277	0.66	1.02	1.3e-01
Top lead variant	2	Breakthrough susceptibility	rs16861415	C	314,073	0.08	0.84	6.8e-55	57,752	0.06	0.89	1.0e-04
Lead variant	2	Breakthrough susceptibility	rs71322420	G	307,474	0.19	0.94	1.2e-15	56,198	0.15	0.97	9.3e-02
Lead variant	2	Breakthrough susceptibility	rs13097481	C	297,587	0.47	1.05	6.0e-13	54,483	0.43	1.03	6.0e-02
Lead variant	2	Breakthrough susceptibility	rs114664200	C	312,314	0.02	0.87	5.6e-09	57,624	0.01	0.95	4.6e-01
Top lead variant	3	Breakthrough susceptibility	rs11673136	G	314,757	0.48	1.08	4.6e-34	58,037	0.46	1.07	1.9e-06
Top lead variant	4	Breakthrough susceptibility	rs112313064	C	305,812	0.36	1.06	1.1e-22	56,617	0.46	1.04	5.4e-03
Lead variant	4	Breakthrough susceptibility	rs3760775	T	312,979	0.06	1.08	3.6e-09	56,541	0.11	1.10	NA
Top lead variant	5	Breakthrough susceptibility	rs681343	T	314,757	0.50	0.95	1.4e-17	58,037	0.45	0.93	1.2e-06
Top lead variant	6	Breakthrough susceptibility	rs1977829	A	311,381	0.18	0.94	1.4e-12	56,989	0.20	0.97	1.1e-01
Top lead variant	7	Breakthrough susceptibility	rs2550250	T	295,060	0.44	1.05	1.8e-12	53,741	0.42	1.06	1.8e-04
Top lead variant	8	Breakthrough susceptibility	rs6676150	C	311,230	0.39	1.04	2.7e-12	56,961	0.38	1.02	1.1e-01
Top lead variant	9	Breakthrough susceptibility	rs17347644	T	310,520	0.35	0.96	1.3e-10	56,818	0.32	0.96	2.1e-02
Top lead variant	10	Breakthrough susceptibility	rs5117	C	312,352	0.24	0.96	4.7e-09	56,918	0.21	0.98	3.8e-01
Top lead variant	1	Breakthrough severity	rs429358	C	74,515	0.15	1.21	1.1e-08	12,758	0.14	1.18	4.7e-02

Supplementary Table 5. Study of the overlap between the different traits. When OR, SE and the P-Value are coloured red, it means that the P-Value is smaller than $5 \cdot 10^{-8}$. SNPs in bold refer to the top lead variants identified in FUMA. Association was tested by logistic regression in REGENIE. **Note:** CHR = Chromosome, OR = Odds Ratio, SE = Standard error, GL = Genomic locus

Type of variant	GL	Phenotype	CHR	SNP	Gene/Nearest gene	Seroconversion						Breakthrough					
						One dose			Two doses			Susceptibility			Severity		
						OR	SE	P Value	OR	SE	P Value	OR	SE	P Value	OR	SE	P Value
**Top lead variant**	**1**	**Seroconversion - One dose**	**6**	**rs9275109**	**HLA-DQB1 - MTCO3P1**	**0.86**	**0.01**	**1.1e-26**	**0.94**	**0.02**	**3.4e-04**	**1.00**	**0.01**	**6.4e-01**	**0.98**	**0.02**	**5.4e-01**
Lead variant	1	Seroconversion - One dose	6	rs3134931	NOTCH4	0.92	0.02	1.4e-08	0.99	0.02	7.4e-01	1.00	0.01	5.7e-01	1.02	0.03	4.0e-01
Lead variant	1	Seroconversion - One dose	6	rs7754570	BTNL2 - HLA-DRA	1.26	0.04	2.6e-08	1.14	0.05	7.8e-03	0.98	0.02	1.8e-01	0.95	0.08	5.0e-01
Lead variant	1	Seroconversion - One dose	6	rs2395195	HLA-DRB9 - HLA-DRB5	0.86	0.02	5.1e-12	0.93	0.02	8.3e-04	0.99	0.01	3.2e-01	0.97	0.04	4.1e-01
Lead variant	1	Seroconversion - One dose	6	rs146281128	HLA-DRB6	0.67	0.07	3.8e-08				1.08	0.03	NA	1.13	0.12	2.8e-01
Lead variant	1	Seroconversion - One dose	6	rs145945003	HLA-DRB1 - HLA-DQA1	1.32	0.04	6.5e-14	1.15	0.04	1.9e-03	0.98	0.02	1.3e-01	1.04	0.07	5.3e-01
Lead variant	1	Seroconversion - One dose	6	rs28802989	HLA-DQA1	0.84	0.02	3.7e-19	0.82	0.02	7.1e-21	1.02	0.01	1.7e-02	1.00	0.03	9.8e-01
Lead variant	1	Seroconversion - One dose	6	rs4713558	HLA-DQA1	0.81	0.03	1.3e-09	0.90	0.04	2.3e-03	0.97	0.01	4.6e-02	0.95	0.06	3.6e-01
Lead variant	1	Seroconversion - One dose	6	rs1794514	HLA-DQB1 - MTCO3P1	1.11	0.02	6.4e-11	1.04	0.02	4.9e-02	1.01	0.01	8.7e-02	0.98	0.03	5.6e-01
Lead variant	1	Seroconversion - One dose	6	rs3129733	XXbac-BPG254F23.7	0.90	0.02	2.1e-08	1.03	0.02	1.4e-01	1.00	0.01	8.0e-01	0.96	0.03	2.1e-01
Lead variant	1	Seroconversion - One dose	6	rs2071472	HLA-DOB - TAP2	0.90	0.02	4.8e-11	0.93	0.02	1.1e-04	1.00	0.01	7.9e-01	1.00	0.03	9.5e-01
Lead variant	1	Seroconversion - One dose	6	rs3130216	COL11A2P1 - HLA-DPB2	0.92	0.01	6.0e-09	0.98	0.02	1.5e-01	1.01	0.01	2.1e-01	0.95	0.02	3.3e-02
**Top lead variant**	**2**	**Seroconversion - One dose**	**2**	**rs79510369**	**PLA2R1**	**1.48**	**0.06**	**5.3e-10**	**1.03**	**0.07**	**7.3e-01**	**0.95**	**0.03**	**1.0e-01**	**1.21**	**0.11**	**9.5e-02**
**Top lead variant**	**1**	**Seroconversion - Two dose**	**6**	**rs68033958**	**HLA-DQB1**	**0.84**	**0.02**	**1.9e-18**	**0.82**	**0.02**	**1.4e-21**	**1.02**	**0.01**	**1.9e-02**	**1.00**	**0.03**	**9.0e-01**
Lead variant	1	Seroconversion - Two dose	6	rs28752480	NCR3 - UQCRHP1	0.97	0.03	2.5e-01	1.23	0.03	1.1e-11	0.99	0.01	3.9e-01	0.90	0.05	3.5e-02
Lead variant	1	Seroconversion - Two dose	6	rs2261033	PRRC2A	0.95	0.01	8.6e-04	0.91	0.02	1.1e-08	1.02	0.01	5.8e-05	0.99	0.02	8.0e-01
Lead variant	1	Seroconversion - Two dose	6	rs732162	BTNL2 - HLA-DRA	1.07	0.01	2.7e-06	1.10	0.02	1.9e-08	0.99	0.01	2.3e-01	0.98	0.03	3.5e-01
Lead variant	1	Seroconversion - Two dose	6	rs28383322	HLA-DRB1 - HLA-DQA1	1.00	0.02	7.9e-01	1.15	0.02	1.8e-11	0.98	0.01	2.4e-03	1.03	0.03	4.2e-01
Lead variant	1	Seroconversion - Two dose	6	rs9275766	XXbac-BPG254F23.7 - HLA-DQB3	0.93	0.02	4.8e-03	1.18	0.03	3.8e-09	0.99	0.01	4.0e-01	0.98	0.04	5.5e-01
**Top lead variant**	**2**	**Seroconversion - Two dose**	**6**	**rs3094055**	**UBQLN1P1**	**1.00**	**0.02**	**7.7e-01**	**0.89**	**0.02**	**2.1e-10**	**1.01**	**0.01**	**1.8e-01**	**0.95**	**0.03**	**5.4e-02**
**Top lead variant**	**1**	**Breakthrough susceptibility**	**3**	**rs73062389**	**SLC6A20**	**0.98**	**0.03**	**4.8e-01**	**1.05**	**0.03**	**1.4e-01**	**1.22**	**0.01**	**4.0e-56**	**0.99**	**0.05**	**8.4e-01**
Lead variant	1	Breakthrough susceptibility	3	rs141045534	LIMD1	1.01	0.07	8.3e-01				1.18	0.03	3.5e-08	0.95	0.11	6.9e-01
Lead variant	1	Breakthrough susceptibility	3	rs59776512	SLC6A20	1.03	0.03	3.6e-01	0.98	0.03	5.8e-01	1.10	0.01	7.1e-16	0.95	0.05	2.7e-01
Lead variant	1	Breakthrough susceptibility	3	rs2531757	SLC6A20 - LZTFL1	0.98	0.02	2.3e-01	0.97	0.02	5.4e-02	1.04	0.01	3.7e-09	0.98	0.03	4.9e-01
Lead variant	1	Breakthrough susceptibility	3	rs6789386	XCR1 - FLT1P1	0.99	0.02	3.8e-01	1.00	0.02	8.6e-01	1.04	0.01	2.7e-08	0.94	0.03	1.3e-02
**Top lead variant**	**2**	**Breakthrough susceptibility**	**3**	**rs16861415**	**ST6GAL1**	**0.98**	**0.03**	**4.2e-01**	**1.01**	**0.03**	**8.5e-01**	**0.84**	**0.01**	**6.8e-55**	**1.04**	**0.05**	**4.1e-01**
Lead variant	2	Breakthrough susceptibility	3	rs71322420	RPS20P14 - RP11-42D20.1	1.02	0.02	4.0e-01	1.02	0.02	2.6e-01	0.94	0.01	1.2e-15	1.03	0.03	3.1e-01
Lead variant	2	Breakthrough susceptibility	3	rs13097481	ST6GAL1	1.03	0.01	5.0e-02	1.00	0.02	9.2e-01	1.05	0.01	6.0e-13	0.99	0.02	7.2e-01
Lead variant	2	Breakthrough susceptibility	3	rs114664200	ST6GAL1	1.03	0.05	5.9e-01	0.97	0.06	6.2e-01	0.87	0.02	5.6e-09	1.03	0.10	7.3e-01
**Top lead variant**	**3**	**Breakthrough susceptibility**	**19**	**rs11673136**	**MUC16**	**1.01**	**0.01**	**6.1e-01**	**1.01**	**0.02**	**4.0e-01**	**1.08**	**0.01**	**4.6e-34**	**0.96**	**0.02**	**7.3e-02**
**Top lead variant**	**4**	**Breakthrough susceptibility**	**19**	**rs112313064**	**FUT6**	**1.01**	**0.01**	**5.2e-01**	**1.01**	**0.02**	**5.9e-01**	**1.06**	**0.01**	**1.1e-22**	**1.03**	**0.03**	**3.0e-01**
Lead variant	4	Breakthrough susceptibility	19	rs3760775	FUT6 - FUT3	0.95	0.03	6.5e-02	1.05	0.03	1.6e-01	1.08	0.01	3.6e-09	1.11	0.05	2.7e-02
**Top lead variant**	**5**	**Breakthrough susceptibility**	**19**	**rs681343**	**FUT2**	**0.96**	**0.01**	**4.4e-03**	**0.98**	**0.02**	**2.7e-01**	**0.95**	**0.01**	**1.4e-17**	**1.05**	**0.02**	**6.8e-02**
**Top lead variant**	**6**	**Breakthrough susceptibility**	**10**	**rs1977829**	**MXI1**	**1.03**	**0.02**	**1.3e-01**	**0.98**	**0.02**	**3.0e-01**	**0.94**	**0.01**	**1.4e-12**	**1.08**	**0.03**	**1.9e-02**
**Top lead variant**	**7**	**Breakthrough susceptibility**	**3**	**rs2550250**	**MUC4**	**0.97**	**0.01**	**6.5e-02**	**1.03**	**0.02**	**1.2e-01**	**1.05**	**0.01**	**1.8e-12**	**0.98**	**0.03**	**3.6e-01**
**Top lead variant**	**8**	**Breakthrough susceptibility**	**1**	**rs6676150**	**HMGN2P18 - KRTCAP2**	**1.02**	**0.01**	**2.6e-01**	**0.99**	**0.02**	**4.8e-01**	**1.04**	**0.01**	**2.7e-12**	**0.98**	**0.02**	**4.1e-01**
**Top lead variant**	**9**	**Breakthrough susceptibility**	**3**	**rs17347644**	**NFKBIZ**	**1.00**	**0.01**	**7.8e-01**	**1.01**	**0.02**	**4.6e-01**	**0.96**	**0.01**	**1.3e-10**	**1.05**	**0.03**	**4.7e-02**
**Top lead variant**	**10**	**Breakthrough susceptibility**	**19**	**rs5117**	**APOC1**	**1.00**	**0.02**	**7.8e-01**	**1.00**	**0.02**	**8.7e-01**	**0.96**	**0.01**	**4.7e-09**	**1.13**	**0.03**	**1.3e-05**
**Top lead variant**	**1**	**Breakthrough severity**	**19**	**rs429358**	**APOE**	**1.00**	**0.02**	**8.5e-01**	**1.02**	**0.02**	**3.5e-01**	**0.97**	**0.01**	**5.3e-04**	**1.21**	**0.03**	**1.1e-08**

Supplementary Table 6. Colocalization analysis of the different traits to study the shared causal variant assumption. If P(H4) is higher than 50%, the N and the P(H4) value are highlighted in red. **Note:** SNP: Single nucleotide polymorphism; CHR = Chromosome; N = Number of variants within ±250kb from the reference variants and hence, used for the colocalization analysis; P(H4): Probability of a shared causal variant.

Phenotype	SNP	CHR	POS	Seroconversion				Breakthrough			
				One dose		Two dose		Susceptibility		Severity	
				N	P(H4) (%)	N	P(H4) (%)	N	P(H4) (%)	N	P(H4) (%)
Seroconversion - One dose	rs9275109	6	32,649,676			11,837	0.00	11,184	0.29	11,702	1.39
Seroconversion - One dose	rs79510369	2	160,858,048			1,877	3.29	1,876	3.60	1,878	5.77
Seroconversion - Two dose	rs68033958	6	32,634,226	11,957	0.00			11,297	5.41	11,815	1.55
Seroconversion - Two dose	rs3094055	6	30,332,146	2,894	0.47			2,884	0.75	2,892	5.30
Breakthrough susceptibility	rs73062389	3	45,835,417	1,633	1.69	1,629	3.55			1,634	2.11
Breakthrough susceptibility	rs16861415	3	186,696,364	1,878	1.64	1,878	1.34			1,880	2.89
Breakthrough susceptibility	rs11673136	19	9,007,748	1,930	0.75	1,927	0.99			1,928	5.13
Breakthrough susceptibility	rs112313064	19	5,831,724	1,486	0.97	1,487	1.52			1,486	23.09
Breakthrough susceptibility	rs681343	19	49,206,462	1,864	26.98	1,869	1.34			1,868	5.31
Breakthrough susceptibility	rs1977829	10	111,975,041	1,330	2.33	1,329	1.55			1,330	12.39
Breakthrough susceptibility	rs2550250	3	195,500,549	1,815	3.49	1,818	2.48			1,820	1.62
Breakthrough susceptibility	rs6676150	1	155,123,837	844	1.30	845	1.00			848	1.61
Breakthrough susceptibility	rs17347644	3	101,547,733	1,495	0.73	1,496	1.00			1,497	5.84
Breakthrough susceptibility	rs5117	19	45,418,790	1,706	0.72	1,701	0.91			1,704	27.58
Breakthrough severity	rs429358	19	45,411,941	1,715	1.00	1,711	2.05	1,708	27.58		

Supplementary table 7. Previously reported variants associated with genomic loci also founded in our study. Association was tested using the respective methods reported in each study. Phenotypes can either be risk of susceptibility or severity of COVID-19.

Genomic loci	Reported	Study/Company	Cases	Controls	Population	Lead variant (Effect allele)	OR (95% CI)	P-Value
SLC6A20	Severe Covid-19 GWAS group[1]	The Severe Covid-19 GWAS group	1,610 (COVID-19 positive, hospitalized with respiratory support)	2,205 (Population controls, unknown COVID-19 status)	Italy, Spain	rs11385942 (GA)	1.77 (1.48, 2.11)	$1.15\cdot10^{-10}$
SLC6A20	Kousathanas et al., 2022[2]	The GenOMICC (Genetics of Mortality in Critical Care) initiative	7,491 (Critically ill patients from ICUs)	48,400 (Controls were from the 100,000 Genomes Project cohort, n = 46,770; and mild COVID-19, n = 1630)	Europeans, South Asians, Africans, East Asians	rs2271616 (T)	1.3 (1.21, 1.37)	$9.9\cdot10^{-10}$
SLC6A20	Thibord F et al., 2022[3]	GRASP portal	16,551 European ancestry cases, 557 African ancestry cases, 810 South Asian ancestry cases, 563 OTHER cases	442,669 European ancestry controls, 7,087 African ancestry controls, 8,607 South Asian ancestry controls, 10,446 OTHER controls.	Mixed	rs73062389 (A)	1.21 (1.15, 1.27)	$4.3\cdot10^{-15}$
SLC6A20	Shelton J., et al., 2021[4]		1,128 European ancestry cases, 245 Hispanic or Latin American cases, 74 Black or African American cases	679,531 European ancestry controls, 94,237 Hispanic or Latin American controls, 22,383 Black or African American controls	Mixed	rs13078854 (G)	0.6 (0.53, 0.67)	$2.0\cdot10^{-18}$
SLC6A20	Shelton J., et al., 2021[4]		9,913 European ancestry cases	85,072 European ancestry controls	Mixed	rs2531743 (G)	0.92 (0.89, 0.95)	$3\cdot10^{-12}$
SLC6A20	COVID-19 Host Genetic Initiative, 2021[5]	The COVID-19 Host Genetics Initiative	13,641 (COVID-19 positive test and hospitalization), 49,562 (Reported SARS-CoV-2 infection), 52,630 (COVID-19 positive), 52,630 (COVID-19 positive), 45,641 (COVID-19 positive and not hospitalized)	2,070,709 (Population-based controls with unknown COVID-19 status), 1,770,206 (Population-based controls with unknown COVID-19 status), 704,016 (COVID-19 negative or unknown), 109,605 (COVID-19 negative), 704,016 (COVID-19 negative or unknown)	Mixed	rs2271616 (T)	1.15	$1.8\cdot10^{-34}$
SLC6A20	Horowitz et al, 2022[6]	AncestryDNA COVID-19 research study, GHS, PMBB, UK Biobank	45,006 European ancestry cases, 2,710 African ancestry cases, 830 South Asian ancestry cases, 174 East Asian ancestry cases, 3,832 Hispanic or Latin American cases	651,358 European ancestry controls, 26,106 African ancestry controls, 10,143 South Asian ancestry controls, 2,779 East Asian ancestry controls, 14,630 Hispanic or Latin American controls	Mixed	rs2531743 (G)	0.96 (0.95, 0.97)	$1.0\cdot10^{-9}$
HLA-B	COVID-19 Host Genetic Initiative, 2022[7]	The COVID-19 Host Genetics Initiative	25,027 (Moderate or severe COVID-19, defined as those hospitalized due to symptoms associated with infection)	2,836,272 (control individuals)	Europeans, Americans, Africans, Middle Eastern, South Asians, East Asians (25 countries)	rs111837807	1.13 (1.09, 1.17)	$2.33\cdot10^{-11}$
HLA-DRB1	Kousathanas et al., 2022[2]	The GenOMICC (Genetics of Mortality in Critical Care) initiative	7,491 (Critically ill patients from ICUs)	48,400 (Controls were from the 100,000 Genomes project cohort, n = 46,770; and mild COVID-19, n = 1,630)	Europeans, South Asians, Africans, East Asians	rs9271609	1.1 (1.09, 1.19)	$3.26\cdot10^{-9}$
FUT2	Kousathanas et al., 2022[2]	The GenOMICC (Genetics of Mortality in Critical Care) initiative	7,491 (Critically ill patients from ICUs)	48,400 (Controls were from the 100,000 Genomes project cohort, n = 46,770; and mild COVID	Europeans, South Asians, Africans, East Asians	rs368565	1.1 (1.1, 1.2)	$3.55\cdot10^{-11}$
FUT2	Kousathanas et al., 2022[2]	The GenOMICC (Genetics of Mortality in Critical Care) initiative	7,491 (Critically ill patients from ICUs)	48,400 (Controls were from the 100,000 Genomes project cohort, n = 46,770; and mild COVID	Europeans, South Asians, Africans, East Asians	rs516246 (T)	0.9 (0.88, 0.93)	$1.4\cdot10^{-15}$
HLA-DQA1	Kousathanas et al., 2022[2]	The GenOMICC (Genetics of Mortality in Critical Care) initiative	7,491 (Critically ill patients from ICUs)	48,400 (Controls were from the 100,000 Genomes project cohort, n = 46,770; and mild COVID	Europeans, South Asians, Africans, East Asians	rs2858305 (T)	0.93 (0.9, 0.95)	$2.1\cdot10^{-9}$
APOE	Thibord F et al., 2022[3]	GRASP portal	16,551 European ancestry cases, 557 African ancestry cases, 810 South Asian ancestry cases, 563 OTHER cases	442,669 European ancestry controls, 7,087 African ancestry controls, 8,607 South Asian ancestry controls, 10,446 OTHER controls.	Mixed	rs429358 (C)	1.40 (1.24, 1.57)	$3.06\cdot10^{-1}$

References

1. D, E. *et al.* Genomewide Association Study of Severe Covid-19 with Respiratory Failure. *New England Journal of Medicine* **383**, (2020).

2. Kousathanas, A. *et al.* Whole-genome sequencing reveals host factors underlying critical COVID-19. *Nature* **607**, (2022).

3. Thibord, F., Chan, M. V., Chen, M. H. & Johnson, A. D. A year of COVID-19 GWAS results from the GRASP portal reveals potential genetic risk factors. *Human Genetics and Genomics Advances* **3**, (2022).

4. Shelton, J. F. *et al.* Trans-ancestry analysis reveals genetic and nongenetic associations with COVID-19 susceptibility and severity. *Nat Genet* **53**, (2021).

5. Niemi, M. E. K. *et al.* Mapping the human genetic architecture of COVID-19. *Nature* **600**, (2021).

6. Horowitz, J. E. *et al.* Genome-wide analysis provides genetic evidence that ACE2 influences COVID-19 risk and yields risk scores associated with severe disease. *Nat Genet* **54**, (2022).

7. Pathak, G. A. *et al.* A first update on mapping the human genetic architecture of COVID-19. *Nature* **608**, E1–E10 (2022).
